# Bibliographical Notices

**Published:** 1846-01

**Authors:** 


### BIBLIOGRAPHICAL NOTICES.

*Animal Chemistry with reference to the Physiology and Pathology of Man.* By Dr. J. Franz Simon, Fellow of the Society for the advancement of Physiological Chemistry, at Berlin, &c. &c. *Translated and Edited* by George E. Day, M. A., and L. M. Cantab. Licentiate of the Royal College of Physicians. Vol. 1, pp. 359, London, 1845. American edition, pp. 292, Philadelphia, 1845.

This is the first volume of one of the excellent works issued by the Sydenham Society of London—an Institution—as we have more than once remarked—worthy of all attention from every one who is desirious of being more than a mere routine practitioner, and of possessing rare works, which it would be impracticable for him to obtain in any other manner. For the trifling annual subscription of *five dollars*, during the last year three treatises have been placed in the hands of the members of the Society in this country, and elsewhere, any one of which is worth the subscription price for the whole. It is surprising, then, under a consideration of these advantages, that the list should not swell beyond all bounds; and if it do not, it must be greatly owing to the objects of the Society not being duly appreciated; and in part to the unobtrusive manner in which its concerns have been, and are conducted. The subscribers ought, indeed, to seek the Society—not the Society the subscribers.

The work of Dr. Simon appeared in Berlin in 1842, and was immediately considered—as Dr. Day has correctly remarked—to be by far the most complete treatise that has appeared on Physiological Chemistry, or, as the Author designated it, "Physiological and Pathological Chemistry of Man," (*Physiologische und Pathologische Anthropochemie.*) Until he became acquainted with the work in 1843, Dr. Day, had entertained the idea of publishing a text book on Medical Chemistry, with the view of attempting to supply a deficiency in the medical literature of his country; but a careful perusal of M. Simon's work convinced him, that he should be doing better service to the profession by undertaking a translation of that work, than the publication of a separate treatise. Under these feelings, he wrote to the author, who immediately offered him all the assistance in his power, and promised him a considerable amount of original matter. His early and unexpected death, however, in the autumn of the same year, rendered the promise "of comparatively little value." "I have, however," says Dr. Day "freely availed myself of the permission granted me by the Council of the Sydenham Society, to insert such additions as the progress of Chemistry, since the original publication of the work, has rendered necessary. These interpolations, with the exception of one class, are distinguished by being included in brackets. I refer to the chemical essays of Simon written with the view of filling up occasional deficiencies in his 'Chemistry of Man,' and published in his 'Beitrage zur Physiologischen und Pathologischen Chemie und Mikroskopie.' This exception was made at the request of Dr. Simon, and its expediency and fairness is [are] unquestionable. The 'Chemistry of Man' was preceded by a volume entitled 'Chemistry of the proximate Constituents of the Animal Body,' which being in fact a distinct work (containing upwards of 500 closely printed pages,) it has been deemed unadvisable to translate in its original form. A brief introduction, in a great measure based upon it, has been drawn up by myself, with the view of facilitating the perusal of the work to those who have not paid much attention to the recent progress of organic chemistry; and having written it with this object, I have intentionally excluded many topics, which some of my readers may consider should have found a place there." (p. iv, Amer. edit.)

A glance at the comprehensive introduction by Dr. Day will show the rapid strides of animal chemistry in recent periods—the very terminology of which must be utterly unintelligible to one who has suffered a few years to elapse in inattention to its progress. We may instance *Protein*, *Pepsin*, *Chondrin*, *Pyin*, *Zomidin*, *Kreatin*, *Hæmaphæin*, *Hæmacyanin*, *Cholepyrrhin* or *biliphæin*, *bilin;* and the different names for the products of the metamorphoses of uric acid.

The first chapter of the 'Chemistry of Man' is on the proximate analysis of compound animal substances. The second—and it occupies the remaining pages of the volume—considers the circulating fluids; and, first of these, the blood. M. Simon first inquires into "*the general physiological Chemistry of the*

*blood*"—in which he includes its general physical and chemical relations,—the development of the blood corpuscles; the phenomena of circulation and respiration; the metamorphoses of the blood, and animal heat. He next examines the special chemistry of the blood—for example, the method of analysing it; the characters of healthy blood, and afterwards those of the diseased fluid.

The chapter on diseased blood is especially full and important. The interesting facts, observed by MM. Andral and Gavarret, from the analysis of the blood in disease are fully stated, with the results of M. Simon's own analysis. Dr. Day, too, has referred to the recent researches of MM. Becquerel and Rodier and others. The analysis of the first named gentlemen were found to correspond generally speaking with those of M. Simon. He affirms, however, that they usually assign a higher proportion to the corpuscles—especially in the blood during inflammatory diseases—than he has found,—a difference, which, he conceives, may be caused by the different methods of analysis pursued by the French observers and himself.

Under this head Dr. Day, refers to the conclusions of MM. Becquerel and Rodier, in regard to the effect of venesection on the relative proportion of the constituents of the blood. Only one of these, however, we shall extract.

*Mean composition of the blood of ten persons bled three times.*

| | 1st. Venesection. | 2d. Venesection. | 3d. Venesection. |
|---|---|---|---|
| Density of defibrinated blood, | 1056.6 | 1053.0 | 1049.6 |
| Density of serum, - | 1028.8 | 1026.3 | 1025.6 |
| Water, - - - | 793.0 | 807.7 | 833.1 |
| Solid residue, - - | 207.0 | 192.3 | 176.9 |
| Fibrin, - - - | 3.5 | 3.8 | 3.4 |
| Albumen, - - - | 65.0 | 63.7 | 64.6 |
| Blood corpuscles, - - | 129.2 | 116.3 | 99.2 |
| Extractive matter and salts, | 7.7 | 6.9 | 8.0 |
| Fat, - - - | 1.662 | 1.584 | 1.530 |
| Consisting of serolin, - | 0.026 | 0.088 | 0.012 |
| " phosphorized fat, | 0.637 | 0.489 | 0.450 |
| " cholesterin fat, | 0.106 | 0.156 | 0.149 |
| " saponified fat, | 0.893 | 0.851 | 0.919 |

From the results of all their observations, MM. Becquerel and Rodier deduce the following conclusions: "In proportion to the number of venesections the blood becomes impoverished and more watery; hence the fall in the density of the defibrinated blood. The albumen diminishes, but only slightly; hence the density of the serum is not much affected. The fibrin is quite uninfluenced by venesection, and its amount is determined by the nature and intensity of the disease. The extractive matters and salts are unaltered. There is a slight diminution in the amount of fat. The various salts are unaffected, and the iron in consequence of its relationship to the corpuscles is diminished. In short, the effect of venesection is to cause a great diminution of the corpuscles, whilst it only slightly lessens the amount of albumen." p. 206.

Useful practical inferences might be drawn from these conclusions. In the first place, we see that where much loss of blood occurs, the blood becomes more watery; hence repeated recurrence of hemorrhage may lay the foundation for its continuance;—especially if water or thin fluids be freely allowed. As the vessels become drained of blood absorption is more active, and if water be taken freely it will speedily pass into the blood-vessels. Hence, to allay thirst in hemorrhage, ice should be taken into the mouth;—or if this should not be at hand, very small quantities of cold water should be allowed for drink.

Again: it would seem that the ratio of the fibrinous element of the blood, which is invariably increased in inflammation, is not reduced by bleeding. On this point MM. Andral and Gavarret accord with these observers,—and it may lead to the inquiry, whether bleeding in the phlegmasiæ have so potent an influence as has been generally, but by no means universally, assigned to it. The observations of Louis in Paris, and of Jackson in Boston, have raised well founded doubts, whether—even in pneumonia—it be as effective an agent as most practitioners imagine. By diminishing, however, the ratio of the red corpuscles, it may prevent hyperæmia.

To the first form of diseased blood described by M. Simon he gives the name *hyperinosis*, from ὑπερ, "in excess," and ἰς, ἰνος, "the fibre of flesh." The chemical and physical characters of the blood in this state are said to be as follows:—

"The blood contains more fibrin than in the normal state, and the corpuscles decrease in proportion to the excess of brfiin ; the fat is also increased. In proportion to the increase of the fibrin and the fat, and the decrease of the corpuscles, the whole solid residue will be diminished.

"The blood coagulates more slowly than in the normal state; the clot is usually not small, but very firm and consistent, and does not break up for a considerable time. It is almost invariably covered with a true buffy coat, (which is produced by the sinking of the corpuscles before the occurrence of coagulation, and by the subsequent coagulation of the fibrin in the layer of serum ) This buffy coat is firm, tough, and intimately connected with the clot; its edge is often turned upwards and its surface uneven. If the clot is small, the buffy coat and the surface of the clot are more or less cupped; the serum is of a pure lemon colour, not tinged red. When subjected to whipping, the fibrin separates in thicker and more solid masses than in ordinary blood. After the removal of the fibrin the corpuscles quickly sink, and frequently occupy only one-fourth of the whole fluid, while in healthy blood they sink very imperfectly, or not at all. The blood has always an alkaline reaction, and is of a higher temperature than in the ordinary state. Lauer found the temperature of the blood in pneumonia as high as 100°, and in bronchitis it reached 101°.6. These temperatures are, however, not higher than are met with in healthy blood. According to Becquerel, the temperature may rise to 5°.4 in inflammatory disease and fevers. According to Goupil, it amounts, in inflammatory disorders, to 106—111°.7, and at the inflamed region to 112°.4.

"The microscope has not yet succeeded in detecting any constant peculiarities.

"The blood occurs in a state of hyperinosis in all inflammatory disorders (Phlogoses.)

"In proportion to the firmness of the clot, the concavity of its surface, (the cupping,) and the toughness and thickness of the buffy coat, is the degree of inflammation; and, conversely, the thinner and more friable the clot is, the less intense is the disorder. We also find, accompanying these physical symptoms, an excess of fibrin, and a diminution of hæmato-globulin, as well as of the solid constituents of the blood generally, and in proportion to the degree in which these phenomena are observed, we may infer a greater or lesser amount of inflammatory action." p. 207.

Analyses of the blood in various hyperinotic affections are then given,—for example in metro-phlebitis puerperalis, carditis, pericarditis with effusion, bronchitis, pneumonia, pleuritis, amygdalitis, hepatitis, lienitis, peritonitis, nephritis and cystitis, rheumatismus acutus, erysipelas, phthisis tuberculosa, febris puerperalis, eclampsia, and carcinoma medullare colli uteri. It may admit of a question, however, whether there be not some error in the observations in regard to the two last affections, which, by the way, are not in M. Simon's book, but have been added by Dr. Day on the authority of Heller. The blood of a girl, subject to convulsions, afforded at one bleeding 6 per 1000 of fibrin—twice the usual proportion; and at another 4.44; another, a case of convulsions, occurring after delivery—gave 5.87 as the ratio of fibrin; but it is proper to remark, that there were concomitant symptoms of metro-peritonitis and endo-metritis, which may account for the increase of the fibrinous element. The most singular case is that of carcinoma medullare colli uteri; the blood of which yielded 16.41 as the ratio of fibrin, and only 77.03 of corpuscles—being an enormous increase of fibrin and a great diminution of the corpuscles. The residue of the serum was almost normal.

M. Simon then passes to the consideration of his second form of diseased blood—*hypinosis*, derived from ὑπο, "under," and ἰς, ἰνος, "fibre of flesh," in which there is a diminished proportion of the fibrinous element; or if it amounts to the normal quantity, its proportion to the blood-corpuscles is less than is found in health, (2.1: 100—Simon, or 3: 110—Lecanu.) The quantity of corpuscles is either absolutely increased, or their proportion to the fibrin is larger than in healthy blood: the quantity of solid constituents is also frequently larger than in the normal fluid. "The clot is commonly large, (but sometimes small,) soft, diffluent, and of a dark, almost black red colour; occasionally no clot is formed. The buffy coat is seldom seen, and when it does occur it is thin and soft, or forms a gelatinous parti-coloured deposit on the clot. The serum is sometimes of a deep yellow tinge, from the colouring matter of the bile, or red from blood-corpuscles in suspension: the blood has always an alkaline reaction. From the numerous analyses of Andral and Gavarret, and from the observations of others, it appears that the blood occurs in a state of hypinosis in fever: if, however, the reaction assumes the synochal type, or if inflammation of the respiratory or other organs supervene, then the fibrin will increase, in a corresponding degree, and the blood corpuscles decrease, so that the blood will approximate in its constitution to the normal standard, or even partially assume the characters of hyperinosis." (p. 236, Am. edit.

Analyses of the blood in various hypinotic affections are then given—in typhus abdominalis, febris continua, variola et morbi varioloides, rubeola, scarlatina, febris intermittens and hæmorrhagia cerebralis.

The chemical and physical characters of M. Simon's third form of diseased blood, *spanæmia,* from σπανος, "poor," and αιμα, "blood," are as follows:—

"The amount of fibrin and of corpuscles is diminished: the amount of residue of serum is either normal or diminished: the proportion of water is higher than in healthy blood: the amount of salts in the serum is sometimes normal, sometimes diminished. The blood is very fluid; it is sometimes of a dark or even violet, and sometimes of a bright colour: it usually coagulates imperfectly, sometimes not at all. The clot is small, soft, diffluent, and neither covered with a true nor false buffy coat. The serum is generally of a bright colour, but sometimes of a dark yellow, or even red tint. The specific gravity of the blood is considerably diminished." (pp. 251, Am. edit.)

Analyses of the blood in various spanæmic diseases are then given: viz., in anæmia and hydræmia, carcinoma, scrofulosis, chlorosis, scorbutus, morbus maculosus Werlhofii, hemorrhages, typhus petechialis putridus, yellow fever, plague.

M. Simon's fourth form of diseased blood, *heterochymeusis,* from ἑτεροσ, 'other,' and χυμευσις, 'mixture,' comprises all those states of the circulating fluid in which a substance is present that does not exist in it normally—for instance, urea in appreciable quantity, sugar, colouring matter of the bile, fat or pus. Analyses are given of blood containing uræa *(uræmia)*; sugar *(melitæmia*; bile *(cholæmia)*; fat *(piarhæmia)*; pus *(pyohæmia)*; and animals. In a supplement, are analyses of the blood of the pregnant female, of the menstrual fluid, and of the blood of animals. The whole section on the blood is exceedingly rich in details, which we may in vain look for in any similar work on the subject.

The organic chemistry of the lymph and the chyle concludes the volume. From all the analyses, Dr. Day infers, that "the lymph is a dilute serum, and the salts of the blood which make their escape along with the colourless *liquor sanguinis* from the capillaries, either return again in the same proportions to each other as they were secreted, into the capillaries, or—which is most probable, they only penetrate into the lymphatic vessels. Besides there being more water in the lymph than in the serum (in the ratio of 950 to 922) the two fluids differ in the ratio of their solid constituents to the salts: in the lymph the salts amount to 11.22, and in the serum to 9.65 per cent. of the solid residue. It is probably this circumstance that causes the much greater viscidity of the serum, which is by no means solely dependent on the larger quantity of albumen in solution." (pp. 288. Am. edit.)

On the chyle the author affords no new information as the result of his own researches.

It will be seen, then, that the work of M. Simon is a valuable addition to the library of the practitioner and student. For the remaining volume we look forward with the greater interest from a knowledge of the excellent contributions to our knowledge of chemical physiology and pathology contained in this. We are not amongst those Utopians who believe, that every material addition to our knowledge of living matter is to be derived from chemistry; yet we feel that every process, healthy or morbid, that takes place in the system of nutrition, is but a form of chemical metamorphosis, controlled, however, by forces, which we may never be able to estimate rightly. Organic chemistry and microscopy are—as they have already been—the most important helpmates to us in our investigation of living acts; and assuredly no one can be esteemed informed on those acts, who is ignorant of the materials contained in the excellent work now under notice.

---

*The Principles and Practice of Obstetric Medicine and Surgery, in reference to the process of parturition. Illustrated by one hundred and forty-eight figures.* By FRANCIS H. RAMSBOTHAM, M. D., etc. etc. *A new edition, from the enlarged and revised London edition.* Royal 8vo. pp. 519. Lea & Blanchard, 1845.

*The Theory and Practice of Midwifery.* By FLEETWOOD CHURCHILL, M. D., etc. etc. With notes and additions by ROBERT M. HUSTON, M. D., etc. etc. *Second American edition, with one hundred and twenty-eight illustrations from drawings by Bagg and others.* Engraved by Gilbert. 8vo. pp. 525. Lea & Blanchard: Philadelphia, 1846.

The extensive distribution of a former edition of each of these standard works among the profession in this country, renders it unnecessary for us to say anything of their general merits. The call for new editions in so brief a space of time, (two years) is sufficiently indicative of the estimation in which they are held, and renders it unnecessary for us to do more than point out the additions and alterations to be found in the present editions; and to these we shall confine our remarks.

The second or revised edition of Dr. Ramsbotham's work contains "some trivial alterations and additions to the original text;" and a short Essay has been added "on the Diseases of the Pregnant and Puerperal States, and on the interesting subject of Abortion. Some statistical tables, afforded by the practice of the Royal Maternity Charity, will also be found appended."

Under the head of "Diseases of Pregnancy," Dr. Ramsbotham gives very clear and judicious directions for the "general management of a pregnant woman," which, if properly attended to, would save a great deal of the suffering that many experience as the penalty of their imprudence or thoughtless neglect. They are of course *hygienic*, and enjoin the avoidance of "every cause of mental excitement, as well as personal fatigue, such as late hours, heated rooms, large assemblies, balls and plays; for in the irritable state of the nervous system induced by pregnancy, slight causes will sometimes produce the most disastrous consequences: thus the mere mental agitation consequent on seeing a deep tragedy might be sufficient to produce abortion. Exercise should be enjoined, short of fatigue, just sufficient to keep the patient in a state of general health. Her food should be simple, and that which is likely to be most easily digested."

"Most of the diseases of pregnancy," the author remarks, "are the consequences either of pressure, distension, inflammatory action, specific contagion, or a peculiar irritation attendant and dependent on the state of pregnancy." Under each of these heads we find appropriate remarks in reference to pathology and treatment.

Speaking of the diseases induced by pressure, the author observes among other things, that "palpitations of the heart are most frequent during the latter months of pregnancy, whilst the uterus is pressing the diaphragm upwards. Syncope, on the contrary, is more often met with during the early months of gestation, and is sometimes very dangerous, especially to the child, since the fœtus is likely to die from the supply of blood to the uterus being suspended during the continuance of the faint: abortion is then consequent on the death of the ovum."

It appears to us that these remarks are calculated, without further qualification, to cause unnecessary alarm to the young practitioner. According to our experience, fainting during the early period of pregnancy, unless attended by some unusual circumstance, as lesion of the heart or large vessels, effusion into the cavities, great debility, or when caused by fright or accident, rarely causes the disastrous consequences spoken of. It is probable that in a vast majority of the cases that occur, the circulation is not entirely suspended during the faint, even for a short period, and hence no evil results to the fœtus—in fact, when the woman recovers, and abortion follows the spell, we are inclined to believe that the ovum is injured by the unwonted activity of the circulation which occurs in the reaction following the fainting fit, rather than from the failure of a supply of blood; at other times, contraction of the uterus is caused by the nervous derangement, and thus destroys the connection between it and the ovum. In one or the other of these modes we apprehend abortion is produced much more frequently than in the way suggested by the author: but, as we have said, where there is no unusual or violent cause operating to induce or complicate syncope in early pregnancy, bad results are not apt to follow. In saying thus much, however, we do not the less deprecate every thing calculated to produce fainting, knowing as we do that danger *may* follow in one or other of the ways pointed out.

Remarking on a varicose state of the veins caused by the pressure of the gravid uterus, Dr. R. says: "Sometimes the veins in the leg will burst, immediate danger is induced, and a troublesome ulcer is eventually left. Death has happened suddenly from such an accident." "Sudden death" surely does not happen from the "troublesome ulcer," but must result from the hemorrhage occurring from the ruptured vein, and as this always occurs in those least supported, or, in other words, in superficial veins, we think sudden death ought to be prevented by any one present having an eye to see and a finger to make compression.

Most of the diseases incident to pregnancy are embraced in this part of Dr. Ramsbotham's work, and are treated of with much ability; our space will not allow us to do more than notice some of the points of interest, and particularly such as appear to us to be presented in a light peculiar to the author.

The diseases of the puerperal state are also considered, some of them at much length. Of these, puerperal fever must be regarded as the most interesting, from the great mortality which so frequently attends it, and on this account, more than its frequency, or than anything peculiar in our author's views, we shall cite his opinions on some of the prominent points in its consideration.

"Puerperal peritonitis," Dr. R. remarks, "appears under two very distinct forms—the one sporadic, in which the cases are isolated, and the disease does not extend; the other, epidemic or contagious; and it is principally to the latter variety that the term of puerperal fever has been applied. I have no doubt myself of the contagious nature of peritonitis, in some instances; I have known it many times both spread through a particular district, and be confined to the practice of a particular person, almost every patient attended by such person, under labour for some time, being attacked, while the neighbouring practitioners did not meet with a single case. It seems capable of being carried from one to another, not only by the ordinary modes in which contagion is usually communicated, but through the dress of the attendants also; so that medical men, nurses, or even visitors, may themselves be the instruments of its fatal propagation from one puerperal woman to others, while their own health is not in the least degree disturbed." (p. 416—17.)

In this strong expression of his opinion of the contagiousness of puerperal peritonitis, our author will find some eminent men disagree with him; but certainly the majority of facts and the weight of opinion among those who have seen most of the disease, of later years, preponderate on his side of the question.

"A question has arisen, and been the subject of much dispute, whether the inflammation which constitutes puerperal peritonitis is of the ordinary kind, or specific and peculiar. Some have supposed that it is nothing more than violent common inflammation, (Mackintosh, Campbell;) others that it is allied to hospital gangrene, (Cruveilhier, Ferguson;) and others, that it is of an erysipelatous character, (Gorden, Louder, Hull, Lee, &c.) The sporadic variety indeed appears to possess no characteristics which could separate it from acute peritonitis occurring in other states of the system; but numerous facts have been published which would lead to the belief that the epidemic form partakes largely of the nature of erysipelas; and I am inclined to this opinion myself." In confirmation of this opinion he refers to the reports of Ceely of Aylesbury, and Ingleby of Birmingham. In regard to the causes of the disease, predisposing and exciting, our author seems in doubt, except as to its occasional origin from contagion.

On the subject of depletion in the treatment of the disease, we are presented with the following judicious observations:

"Every one must acknowledge that this subject is beset with great difficulties, both in description and practice; and these difficulties are increased by the fact that the various epidemics, whose history we possess, have differed much from each other, being modified by the constitution of the atmosphere, and partaking much of the character of the then prevailing diseases. What Sydenham has designated the *constitution of the year*, has scarcely been taken into account in the treatment of these diseases; this, however, it is of the utmost importance to attend to; for we shall invariably find that if the common fevers of the season bear depleting well, the same means will prove efficacious in arresting the puerperal diseases at the same time; while, on the other hand, if the typhoid type prevail, the lancet must be employed with a more sparing hand."

Acute tympanitis is very well described, with its appropriate treatment, and the diagnosis of the affection as compared with peritonitis, and the importance of the distinction in the management of each, are clearly set forth.

Typhus, occurring during the puerperal state, is also spoken of, although briefly. According to the author's experience, it "is very rare in the puerperal state—the most uncommon of all the affections which have been described under this denomination." "To this the term *puerperal fever* would be peculiarly proper, and might be retained if it had not already been employed in so vague and undefined a sense." The description of it is in fact that of the more malignant forms of puerperal fever, as described by many writers under the title of puerperal peritonitis.

The present edition contains several notes and an appendix, omitted in the previous American edition. The latter comprises some interesting historical notices and useful remarks in relation to *ergot, the forceps, transfusion,* &c. The observations on ergot contain a succinct history of its introduction as an article of the materia medica, the various reports and opinions of those who first employed it, with occasional comments on the views entertained in regard to its safety and efficacy in promoting parturient action. In connection with the latter point, we find our own opinions referred to, in a way that leads us to think our expressions have been misapprehended. "Again," remarks our author," it was objected that the medicine, if commonly introduced into practice, would be dangerous, because it might be given in cases perfectly unfitted for its use, (Huston, North Amer. Med. Surg. Journ., January, 1829;) and that contusions, inflammations, sloughings, and lacerations of the uterus, vagina, and perineum, would frequently follow its injudicious employment. It surely is neither sound logic nor fair argument to adduce as an objection against a valuable remedy, the possibility of its abuse. I would ask, is bleeding never to be abused, or mercury? And shall we discard these remedies because a bungler might misapply them? Neither is this or any other medicine to be prescribed at random; we must only have recourse to it in consequence of certain conclusions at which our mind has arrived after a system of severe reasoning."

The argument in this extract is logical enough, but we deny its application to us. The catalogue of injuries enumerated by the author, we believe is not contained in the essay referred to, although we are free to admit that the arguments against the general employment of the article are pushed very far—possibly farther than experience will justify. It happens on the first introduction of a new remedy in the treatment of disease, especially if sustained by eminent authorities, that every body is agog to try it—suitable cases for its use are found every day and by every practitioner—after a while, oft repeated disappointment corrects the enthusiasm, and it sinks to its proper level in importance, and sometimes below it. Such was the case with ergot when our paper was read before the College of Physicians. The too indiscriminate employment of it had caused consequences so fearful as to awaken the attention of even the lay members of the community, and the daily newspapers were loud on the subject of the number of the still-born which appeared in the weekly reports of our Board of Health. It was under these circumstances that we endeavoured to show to the profession the dangerous character of the agent they were so unscrupulously employing, and that when given in the doses and under the circumstances prescribed by eminent teachers, disappointment and mishaps would occasionally follow. We are gratified in believing that our opinions on these points have since been shared in by the experienced practitioners generally through the United States, and that the use of the article is now greatly restricted.

The profession is under great obligations to the pnblishers for the manner in which the valuable work of Dr. Ramsbotham is brought out. Thick white paper, excellent type and magnificently executed illustrations, like those in the volume before us, are something to enjoy in this day of cheap publishing.

The writings of Dr. Churchill are so familiar to American physicians that little need be said in reference to the work before us. Comprehending all the subjects properly belonging to obstetrics, it is written with such conciseness as to bring the whole within the compass of a moderate sized octavo. Eminently eclectic as a writer, the author is always impartial in his search after materials for his work, and judicious in their selection. So little time has elapsed since the former editions came from the press, that it can hardly be expected that much has occurred to be added to the present; nevertheless, the editor has found occasion to introduce some interesting observations on physiological and practical points of great importance. In physiology, especially, the labours of recent inquirers have added most to our knowledge, and it is to points of this character, connected with obstetrics, that much of what is added relates. On the subjects of turning, management of cases of placenta prævia, and one or two other points, the practice of the art has experienced some modifications. On all these subjects the editor has endeavoured to make the present edition a faithful exposition of the existing state of the science.

*On Diseases of the Liver.* By George Budd, M. D., F. R. S., Professor of Medicine in King's College, London; and Fellow of Caius College, Cambridge. *With coloured plates and numerous wood-cuts.* 8vo. pp. 392. Lea & Blanchard, Philadelphia, 1846.

The materials of which the present volume is composed accumulated gradually during eight years in which the author was engaged in hospital practice. For the first three of these years he was the visiting physician to the Seamen's Hospital, Dreadnought, where his attention was especially called to diseases of the liver, which are there very frequent among men who have been much in India and other hot climates.

In temperate climates, the prevalence of affections of this viscus has been greatly overrated; and many diseases have been regarded as hepatic or bilious, which, if they implicate the liver at all, do so indirectly. In by far the majority of cases the mischief is gastro-enteric,—the morbid action in the mucous lining of the stomach and intestines extending by continuous sympathy to the liver and gall-bladder. Constantly, indeed, an individual was formerly said to be "bilious," or his liver was affirmed to be out of order, if he suffered from the ordinary results of indigestible aliment; and it was probably with such belief that certain articles of diet—as milk—were esteemed "bilious,"—by the term nothing more being understood, than that they gave rise to phenomena of indigestion, which phenomena were considered to be bilious. We well recollect, some years ago, seeing a case of what appeared to us to be ordinary ophthalmia, which had been pronounced to be owing to some morbid condition of the liver and had been unmercifully treated by the internal use of the great "antibilious" agent—calomel.

Again, how vague were our former notions in regard to the therapeutics of "liver disease." A mere routine system was generally pursued, and the "Sampson article of the materia medica," as our Southern friends term calomel, was thrown into the economy, under the vague notion of its being able to search out for the enemy; or rather to discover whether such enemy were really present and in ambush. If suspicion existed that bile was the offender, then *it* must be expelled, and what cholagogue could be equal to calomel or blue pill!—what so efficacious—to use the language of either Stokes

3*

or Graves—we do not recollect which—as "a blue pill at night, and a pot of fæces in the morning"!

Yet these mercurial cathartics are really good cholagogues. It may well be questioned, however, whether this effect be induced by any special action exerted by them upon the liver—that is, when they are given as purgatives. They are agents that affect by preference the upper portion of the intestinal tube, as we know other cathartic agents do the lower, and whose operation is, therefore, necessarily extended along the gall ducts, so as to excite the liver to greater secretion. We have never had sufficient reason for the belief, that mercury—when received into the system, so as to produce any marked agency—expends its action upon the hepatic more than upon any other organs of the secretory system.

Of late years, organic diseases of the liver have really been with us uncommon. This has doubtless been mainly owing to the temperance reformation. A great and manifest cause of such diseases is the use of alcoholic liquors, which pass readily into the veins of the stomach by imbibition, reach the liver through the blood of the vena porta, and cannot fail to modify the nutrition of that gland, so as to occasion marked organic changes, which have even received their names from the presumed cause. It was to the salutary influence exerted by this reformation on the lower classes of society, that Dr. Dunglison—we believe with propriety—in a clinical lecture, published in this journal, ascribed his inability to exhibit before the clinical class during a whole winter at the Philadelphia Almshouse Hospital a single case of liver affection. We fear, however, from the signs of the times, that these improvements in the habits of the people are not likely to be permanent; and that we may be doomed to see again many of those morbid conditions that had become unfrequent; and hence any work, from approved authority, on Liver Diseases, must be hailed and received with satisfaction.

In the introduction to his monograph, Dr. Budd gives a clear exposition of the present views in regard to the structure and functions of the liver—the character and uses of the bile; with some observations on cholagogue agents not characterized by extraordinary lucidity. He is of opinion that taraxacum has undoubtedly

"the effect of rendering the liver more active, and increasing in this way the secretion of bile"—which we think more than questionable, or at least we would say—*quod est demonstrandum.* As undoubtedly he thinks iodine and muriate of ammonia have a similar agency. It may be so; but we want the proof. "Pepper, ginger, and other hot spices," he adds, "are also supposed, perhaps justly, to render the liver more active, and increase the secretion of bile. The great relish with which they are eaten by our countrymen in the East and West Indies, gives considerable sanction to this opinion." p. 96. This we regard as a *non sequitur.* Why may not the effect of these agents be exerted solely or mainly upon the stomach; and where is the *positive* evidence that the secretion of bile is increased by them?

He considers that "most purgatives, but especially rhubarb, have perhaps an effect of the same kind, and may fitly be styled, in the language of our fathers, *cholagogues.*" Yet, we have no reason—from our own observation assuredly—for the belief, that rhubarb is entitled to special mention as a cholagogue: the qualifying word "perhaps," shows, indeed, that Dr. Budd himself is not prepared to speak confidently on the subject from his own experience.

The chapters on Congestion of the Liver, Inflammatory Diseases of the Liver, Diseases which result from faulty nutrition of the liver, or faulty secretion; and on those that are owing to some growth foreign to the natural structure—as well as that on jaundice—contain matter of much interest ably treated; but it is impossible for us to go into a critical or extended notice of them. Dr. Budd is generally well informed as to what has been done by others, and has added the results of his own personal observation.

The appendix contains an account of the liver fluke—*distoma hepaticum;* its effects on sheep and other graminivorous animals; with accounts of some of those entozoa, found in the gall-ducts, duodenum and branches of the portal vein in man.

The publishers have brought out this American edition in a creditable manner as regards the typography, paper and illustrations; and, on the whole, we can recommend "Budd on Diseases of the Liver" as one of the most interesting and complete monographs on the pathology of that important organ that we have seen. It is clearly the production of a gentleman and a scholar.

*A Pocket Atlas of the Descriptive Anatomy of the Human Body*, by J. N. Masse, M. D., Professor of Anatomy, Paris. *Translated from the last Paris Edition and edited by* Granville Sharp Pattison, M. D., Professor of Anatomy in the University of New York, etc., etc. 12 mo. Harper & Brothers, New York, 1845.

The object of the author in preparing the work before us does not seem to have been to supply any deficiency in the number or completeness of existing Anatomical Atlases, but to offer to the student one adapted by its size to be carried in his pocket and used in the dissecting room. There can be no doubt of the convenience of such a work, provided it be sufficiently clear and comprehensive to direct the inquirer in all the points necessary for him to investigate. There is much difficulty, however, in conveying accurate notions of the various parts of the body, and their proper relations, by even the best executed drawings, and when the engraved copies of such drawings are on a greatly reduced scale, the difficulty is proportionally increased. Much depends, too, on the skill exhibited in choosing the best position in which to show the parts to be displayed to the best advantage, and in crowding no more into a picture than is necessary to give correct ideas of the relation of parts. The value of a picture may be greatly diminished by representing too much in a given space, and a principal object may be obscured by attempting to show others of little importance. We have not had time to examine the plates of Masse with sufficient care to be able to say whether they are generally free from these objections. In some instances we have observed that to exhibit one organ another is drawn out of the way, and thus various parts are put upon the stretch in such a manner as to alter materially their relative positions. We have an example of this in plate 47, where the tongue is pulled out of the mouth in order to give a view of the parts collateral or posterior to it, and of course all the parts with which the organ is connected are more or less displaced. A like displacement occurs in plate 55, where the bladder is distended to a degree out of all proportion to the size of the body, with the ureter dangling at its side; and so of several other of the figures. Generally speaking, however, the plates are creditable specimens of the arts. In a few instances, as plates 48, 61, &c., they are not entitled to this praise. The translation is well done, and, taken altogether, it is a neat and useful little volume.

*Manual of Diseases of the Skin. From the French of M.M. Cazenave and Schedel, with Notes and Additions, by Thomas H. Burgess, M. D., etc. Revised and corrected, with additional Notes.* By H. D. Bulkley, M. D., Lecturer on Diseases of the Skin, Fellow of the College of Physicians and Surgeons, New York, etc. 12 mo. pp. 341. J. & H. G. Langley, New York, 1846.

If the Profession in this country were unacquainted with the merits of the treatise of M.M. Cazenave and Schedel on the skin, it would be a sufficient commendation to say that it contains the views of the celebrated Biett, of the hospital St. Louis. Several years ago, a translation of the first edition was published in Philadelphia, and, if we are not mistaken, subsequently a second edition of the same. The present publication is a translation of the *third* Parisian edition, admirably executed, and enriched with valuable additions by both the translator and the editor. Dr. Bulkleyhas been an industrious cultivator in the field of dermatology for many years, and his notes bear evidence of success as well as zeal in his inquiries.

Where there is so much to elicit praise, it is with sincere regret that we find occasion of complaint; nevertheless, we cannot avoid the expression of our surprise that so excellent a work, on subjects so obscure, should be put forth without suitable pictorial illustrations. To those who do not make cutaneous diseases special objects of study, the diagnosis, in many cases, is exceedingly difficult. To undertake the study of such complaints from even the best descriptions, without proper drawings, is about as satisfactory as the study of geography without maps.

---

*A Treatise on Corns, Bunions, the Diseases of the Nails, and the general management of the Feet.* By Lewis Durlacher, Surgeon Chiropodist (by special appointment) to the Queen.

This is a re-print of a new work, just out from the London press, and written by one who is evidently skilled in his particular profession.

No other ailments occur so frequently as the class embraced in this treatise, and considering that they rarely involve the general health or life of the afflicted, cause so much suffering, or are so little understood or so unskilfully managed. A short, sensible, and concisely written work, couched in language intelligible to every one, like the present, has been long needed, and it is to be hoped will be generally welcomed. To country practitioners, especially, it must prove exceedingly useful by the ingenious hints and sound instruction it affords in relation to the treatment of these troublesome complaints.

